# Specialist Coaching Integrated into a Department of Methodology in Team Sports Organisations

**DOI:** 10.1186/s40798-020-00284-5

**Published:** 2020-11-16

**Authors:** Fabian W. Otte, Martyn Rothwell, Carl Woods, Keith Davids

**Affiliations:** 1 Institute of Exercise Training and Sport Informatics, Department of Cognitive and Team/Racket Sport Research, German Sport University Cologne, Am Sportpark Müngersdorf 6, 50933 Cologne, Germany; 2 Sheffield Hallam University, Sheffield, UK; 3 Institute for Health and Sport, Victoria University, Melbourne, Australia

**Keywords:** Ecological dynamics, Specialist coaching, Department of Methodology, Australian Football, Association Football goalkeeping, Rugby League

## Abstract

With increasing resources in sports organisations being allocated to the development and preparation of individual athletes and sub-groups with specialist performance roles, the work of coaches, specialist (role) coaches and support staff needs to be functionally and coherently integrated. This integration of sport science support and coaching can be administered by staff in a Department of Methodology (DoM). Particularly, in this paper, we propose how specialist coaching can be situated in a DoM, presenting a model advocating effective functioning in high-performance team sports organisations. Using principles of ecological dynamics, we provide a rationale for a functional methodology for the design of practice tasks in a DoM that views learners as *wayfinders*, self-regulating their way through competitive performance environments. This rationale for athlete self-regulation in practice could improve athlete performance by enhancing problem solving, engagement with constraints of learning designs and supporting better attunement to contextual information abundant in a competitive environment. Finally, by introducing this unified and multidisciplinary DoM, specialist coaches, team coaches and sport science support staff, within the organisational structure, can collaboratively debate and co-design individualised athlete training programmes to enrich skill adaptability and performance functionality. To underline these contentions, three high-performance sport case studies from Australian Football: goalkeeping in Association Football and Rugby League are presented.

## Key Points

- Underpinned by principles of ecological dynamics, this opinion piece proposes a model for how specialist coaching in high-performance team sports organisations can be situated in a Department of Methodology (DoM).
- The presented model illustrates processes for (1) reviewing the athlete development programme and training environment; (2) examining the athlete-support structure and (3) establishing a DoM structure to cohesively guide athlete development strategies.
- To underline all contentions, three high-performance sport case studies from Australian Football; goalkeeping in Association Football and Rugby League are presented.

## Introduction

Recently, Otte et al. [1] proposed that specialist (role) coaching is an area of growing importance in high-performance sport. With increasing resources in sports organisations being allocated to the development and preparation of individual athletes and sub-groups with specialist roles, the work of coaches and support staff needs to be integrated with functional coherence. In athlete development and preparation for performance, specialist coaching is increasingly necessary, in individual and team sports, for males and females, at late development and senior performance levels [2]. Examples of specialist roles in sport include wicket keeping, slip fielding, batting and bowling in cricket, functioning as forwards, props and backs in rugby union and climbing boulders, mountain surfaces or frozen waterfalls in climbing.

Access to sport science support and coaching can be administered by staff in a Department of Methodology (DoM), where service unification is guided by principles of ecological dynamics [3, 4]. Yet, little is known about how coaching and sport science support can be provided for athletes with specialist roles [1]. Here, underpinned by principles of ecological dynamics, we propose how specialist coaching can be situated in a DoM in high-performance team sports organisations.

## A Department of Methodology

The introduction of specialist coaches purported to enhance athlete development in high-performance sport has contributed another layer of complexity to the multidisciplinary nature of performance preparation. Addition of more sub-discipline specialists to an often already large multidisciplinary team of practitioners (e.g. high-performance managers, strength and conditioning specialists, trainers, coaches, sport psychologists, performance analysts and skill acquisition specialists) might actually hinder, rather than enhance, athlete performance. Although this level of expertise may provide an illusion of integration in a sports organisation, sports practitioners have traditionally operated in a non-integrative way, with sub-discipline specialists working in silos, leading to disjointed athlete preparation [5]. Major challenges in such athlete support teams include (in)effective communication, and the potential for confusion, with discipline specialists potentially adopting a variety of engrained philosophies, theories, methods and techniques in applied practice for athlete development and learning.

It has been argued that the DoM concept can alleviate issues associated with disciplinary integration [3]. A DoM is a method of multidisciplinary functioning that draws on the rich empirical and experiential knowledge of a group of practitioners and applied scientists in a democratic fashion (to avoid prioritising one sub-discipline over another), to attend to the fundamental relationship between theory and practice. Indeed, whilst the DoM concept was first proposed by Rothwell et al. [3], there have been calls for greater disciplinary functioning within sport science for the last two decades (for example, see [6–9]). Whilst the nuances of which ‘type’ of disciplinary functioning fall outside of this opinion piece, Rothwell et al. [3] proposed that multidisciplinarity is what could enable practitioners to draw on knowledge from different sub-disciplines, whilst remaining within the bounds of their expertise. Accordingly, this would be the preferred disciplinary functioning within a DoM (as proposed by Rothwell et al. [3]), as in practice, it would likely afford integrative and collaborative workings whilst preserving the fundamentality of each sub-discipline’s service. The aim of a DoM, therefore, is for specialist coaches and support practitioners to work within a unified conceptual framework to (i) coordinate activity through shared theoretical principles and concepts, (ii) communicate coherent ideas, and (iii) collaboratively design practice landscapes rich in information (i.e. visual, acoustic, proprioceptive and haptic) to guide emergence of multi-dimensional behaviours in athlete performance [10].

Next, we discuss how the integration of sub-discipline specialists within a complex system, such as a DoM, can be achieved through the conceptualisation of ecological dynamics. This, we contend, will enable the coordination of common approaches, co-designed principles and language used by specialist coaches to integrate and enhance performance preparation.

### Key Concepts in Ecological Dynamics that can Underpin a Department of Methodology

Ecological dynamics provides a valuable framework for organising continuous collaborations, exchanges and interactions between athletes, coaches and support staff in a DoM [3]. It enables the implementation of a model of the learner, and the learning process, needed to underpin the professional practice of all staff members supporting the development and performance preparation of individual athletes [11]. The conceptualisation underpinning the deep integration of work and interactions between support staff and coaches has been outlined by Rothwell et al. [3] and Ribeiro and colleagues [12]. In ecological dynamics, athletes and teams exemplify *complex adaptive systems* with inherent self-organising tendencies, which support the emergence of synergies (coordinated functional relationships between system components: in performers and teams) guided by contextual information detected from performance environments. The detection of information is important for regulating actions, and the skilled coupling of perception and action throughout the performance is a hallmark of expertise in sport [13]. Another characteristic of complex adaptive systems is the non-linearity of development; therefore, the relationship between time spent in practice and an athlete’s or team’s development is not deterministic. The emergent nature of a complex adaptive system means that small changes in the way an athlete interacts with the environment, due to carefully co-designed practice interventions discussed previously, could have a large effect on the global system (for example, a small improvement in attacking play in team sports can be achieved by all sub-discipline specialists collaborating to attune athletes to relevant affordances, which can have a significant impact on overall team performance).

Over the years, evidence has supported the empowering benefits of a tendency for local organisation of actions between performers and environments, regulated by emergent contextual information from the displacement and orientation of other performers and characteristics of playing areas [14]. Information sources present in performance environments continually invite actions (affordances) from performers. For example, gaps perceived in defensive lines may invite opportunities for attackers to exploit and penetrate in rugby union, whilst the positioning of defenders closer to the hoop may afford more three-point scoring opportunities for an offensive team in basketball. As such, an important challenge for coaches and sport scientists is to coordinate activity and ideas on designing affordance landscapes in practice to challenge individuals and teams, based on performance information [1, 3, 4].

In ecological dynamics, this has been captured within the notion of *representative learning design* [4, 10], a concept describing the importance of practice tasks simulating (i.e., faithfully representing) the informational constraints present in competition. For example, practitioners can design in representative informational constraints (i.e., opposition ball movement strategies in football, the strategic placement of fielders in cricket or the use of a goalkeeper in hockey) to guide the attention of performers towards the perception of opportunities for action (i.e., affordances) that enable the achievement of a task goal. In this sense, practitioners show performers where to look through carefully designed in constraints, but do not tell them what to see through pre-programmed movement solutions [11].

This yields stark contrast to the more traditional means of guiding the formation of synergies in athletes through augmented information prescribed by coaches and sport science support staff (imposed global sources of information driven from ‘top down’) [12]. Feedback and verbal instructions are two informational constraints, which have been over used in sports like football [15, 16]. More recently, Woods and colleagues [11] criticised the over use of traditional prescriptive coaching methods, using the analogy of a global positioning system (GPS) which imposes a navigational route on an individual, suppressing opportunities for *wayfinding*. They argued that a more functional methodology to be adopted in the design of practice tasks in a DoM could be predicated on ecological and social anthropological information, emphasising learners as *wayfinders*, self-regulating their way through competitive performance environments. Specifically, Woods et al. [11] proposed that the learning process in sport is predicated on the continual detection of information that specifies opportunities for action, with performers learning to detect this information as they self-regulate through emergent performance-related challenges designed into practice tasks by practitioners.

This ecological dynamics rationale for athlete self-regulation in practice could improve athlete performance by enhancing decision-making and problem solving, heightening self-awareness and engagement with constraints of learning designs, and supporting better attunement to contextual information abundant in a competitive environment. The integrated and organised activity of a DoM, replete with specialist coaches, could ensure that each athlete is supported and engaged in co-designing practice tasks, based on performance data and athlete self-awareness regarding areas for improvement in training and competition.

In the rest of this opinion piece, case studies from high-performance sports organisations will be used to demonstrate how specialist coaches could contribute to a DoM to develop athletes and prepare them for performance. To instantiate these case studies, we introduce a model that situates and supports the effective functioning of specialist coaches in athlete development and preparation for performance.

### Effective Functioning of a Department of Methodology Including Practitioner Support for Athletes with Specialist Roles

**Fig. 1:**
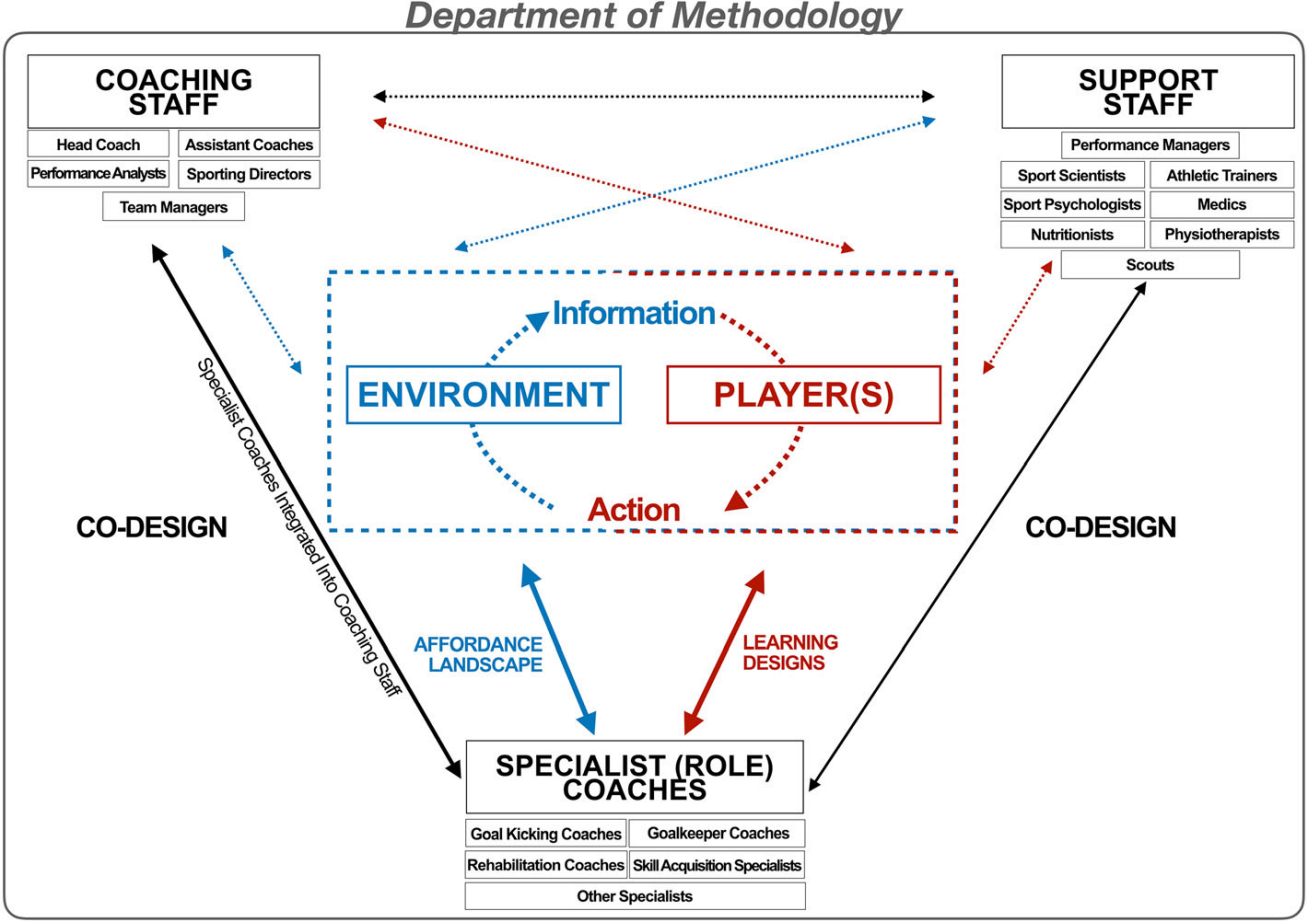
A Department of Methodology model integrating the work of specialist (role) coaches, professional coaching staff members and support staff in order to co-design adaptable learning environments

Through the integration of key concepts in ecological dynamics, the model in Fig. 1 exemplifies the effective functioning of a DoM in high-performance sport. By introducing this unified and multidisciplinary model, specialist coaches, team coaches and sport science support staff can collaboratively debate and co-design individualised athlete training programmes to enrich skill adaptability and performance functionality [2, 3, 5]. These athlete-support structures have the goal of preventing confusion through adoption of contrasting athlete and skill development frameworks (e.g. see [2] for recently presented viewpoints on contrasting ‘traditional’ and ‘contemporary’ skill training approaches). Specifically, they aim to “provide an integrated platform for coaches to collaborate as part of a large group of practitioners, whilst maintaining a coaching focus on individual athletes or sub-groups” ([1], p. 3].

The model in Fig. 1 illustrates (1) reviewing the athlete development programme and training environment (i.e. the dotted box in the centre); (2) examining the athlete-support structure, including co-designing interactions between specialist coaches, coaching and support staff and senior, experienced athletes (i.e. the downward-pointing triangle); and (3) establishing a DoM structure to cohesively guide athlete development strategies (i.e. the frame enclosing the model). First, adhering to principles of a player-environment-centred coaching approach (e.g. see [10]), the constant and reciprocal coupling of information and action within rich affordance landscapes is positioned at the centre of the model. Particularly, coaches’ efforts towards developing variable practice designs that complement individualised athlete development strategies aim to enhance the effective DoM functioning. By designing representative tasks, emergent functional behaviours through holistic integration of various movement-regulating sub-systems, such as perception, action, cognition and emotion, are targeted [1, 2].

Second, the notion that coaches and staff members collaborate to co-design constraints of learning environments for players gains utmost importance [13]. Based on a unified functioning, the communication and exchange of ideas between professional staff members, informed by experienced player perceptions, seeks to support a coherent athlete performance preparation and development methodology within the organisation [1]. Given this open and collaborative communication structure, the coordination and facilitation of role-based and individualised athlete development arguably becomes significantly more accessible and goal-oriented for all professional staff members involved (i.e., in Fig. 1 shown by the three groups of ‘specialist (role) coaches’, ‘coaching staff’ and ‘support staff’ that are connected through numerous arrows).

Third, the encompassing structure of a DoM, adopting a theoretical framework, is proposed to provide fundamental orientation to guide practitioners’ development of individualised learning designs and coaching approaches. Finally, the DoM, as the umbrella constructs for a cohesive and integrated athlete support structure, may effectively exploit opportunities and circumvent challenges for coaches within the organisation (see [1] for an elaboration on opportunities and challenges for specialist coaches). To further underline this argument and highlight the abovementioned tenets of Fig. 1, the following sections offer three high-performance sport coaching case studies of the integration of specialist coaches in a DoM from (1) Australian Football; (2) goalkeeping in Association Football and (3) Rugby League.

## Case Studies

### Case Study 1: A Specialist Goal Kicking Coach Functioning Within a DoM in Elite Australian Football

The premise of Australian Football is to invade territory to score a goal (resulting in six points) by kicking the ball through two goalposts, 6.4 m apart. If the player kicking misses the goal, but the ball still passes between a goal- and point-post (or hits a goalpost), their team will be awarded one point (referred to as a ‘behind’). Scoring can occur both in ‘live play’, or via a ‘set shot’; a task in which a player marks the ball or obtains a free kick, affording them a 30 s unimpeded window to shoot at goal. Critical to this example, goal conversion is an important part of success in the Australian Football League (AFL [17]), with distance and angle to goal being two task constraints that impact the likelihood of successful conversion [18]. It is, therefore, a skill often prioritised in preparation for performance models, typically facilitated by a specialist goal kicking coach. Importantly, a specialist goal kicking coach functioning in a DoM, underpinned by ecological dynamics, would view themselves through the same *learning designer* lens as other coaching and support staff within the DoM. This is integral, as although practice designs may differ between practitioners, they are still bound by the same unifying principles of an ecological dynamics framework (Fig. 1).

The practice task presented in this example was drawn from a professional Australian Football organisation, in which a specialist goal kicking coach aimed to exploit the inherent self-organising tendencies of the players through the promotion of an external focus of attention (i.e., directing a player’s attention towards features ‘outside’ of their body) during the ‘set shot’. The ensuing task, described below, was co-designed between a specialist goal kicking coach and an applied skill acquisition scientist, both functioning within a DoM at the elite Australian Football organisation. This enabled the co-design of a practice task that was both experientially and empirically enriched.

Briefly, the task design required players to (1) perform a ‘set shot’ from distances and angles representative of kicks recorded during game-play (locational information gathered from performance analysts), (2) compete against a teammate of similar skill, with the ‘winner’ being the player first to score > 60 points.

These initial task design features were guided by principles of *representative learning design* [19, 20] and *affective learning design* [21]. To unlock self-organising tendencies within the players and promote an external focus of attention, two additional goalposts were placed within the two larger goalposts, separated by a width of 3 m. The players were instructed that they would receive 12 points if they kicked the ball through the smaller-width goalposts, six points if they kicked a ‘regulation’ goal (i.e., the ball passes between the smaller ‘added’ goalpost and regular goalpost), and one point if they kicked a ‘behind’. Last, if the goals were missed entirely, six points were deducted from a cumulative total—inducing risk and emotion into the learning design. As the task initiated, the specialist coach adopted a ‘hands off’ approach, allowing the players to continually self-regulate perceptions, actions, cognitions and emotions, working to exploit the task constraints to achieve the task goal (i.e., outscoring their opponent by maximising points scored through accurately kicking at goal).

There are two important practical implications to highlight in this case study to exemplify the effective functioning of a specialist coach in a DoM. First, the specialist goal kicking coach functioned cooperatively with a skill acquisition specialist to facilitate *wayfinding* [11], co-designing a practice task that was both experientially and empirically enriched. Second, no specific instructions were given to the players completing the task about how to perform a kick (i.e., mechanistically derived ‘techniques’ or behavioural patterns), being free to explore their own ‘ways’ of goal kicking during the ‘set shot’. Additionally, other than the use of the (smaller added) goalposts, no explicit target was used to prompt the players' attention, with them being free to settle on a target beyond the goalposts they felt was attainable based on their perceived action capabilities.

### Case Study 2: Goalkeeping in Association Football

**Fig. 2:**
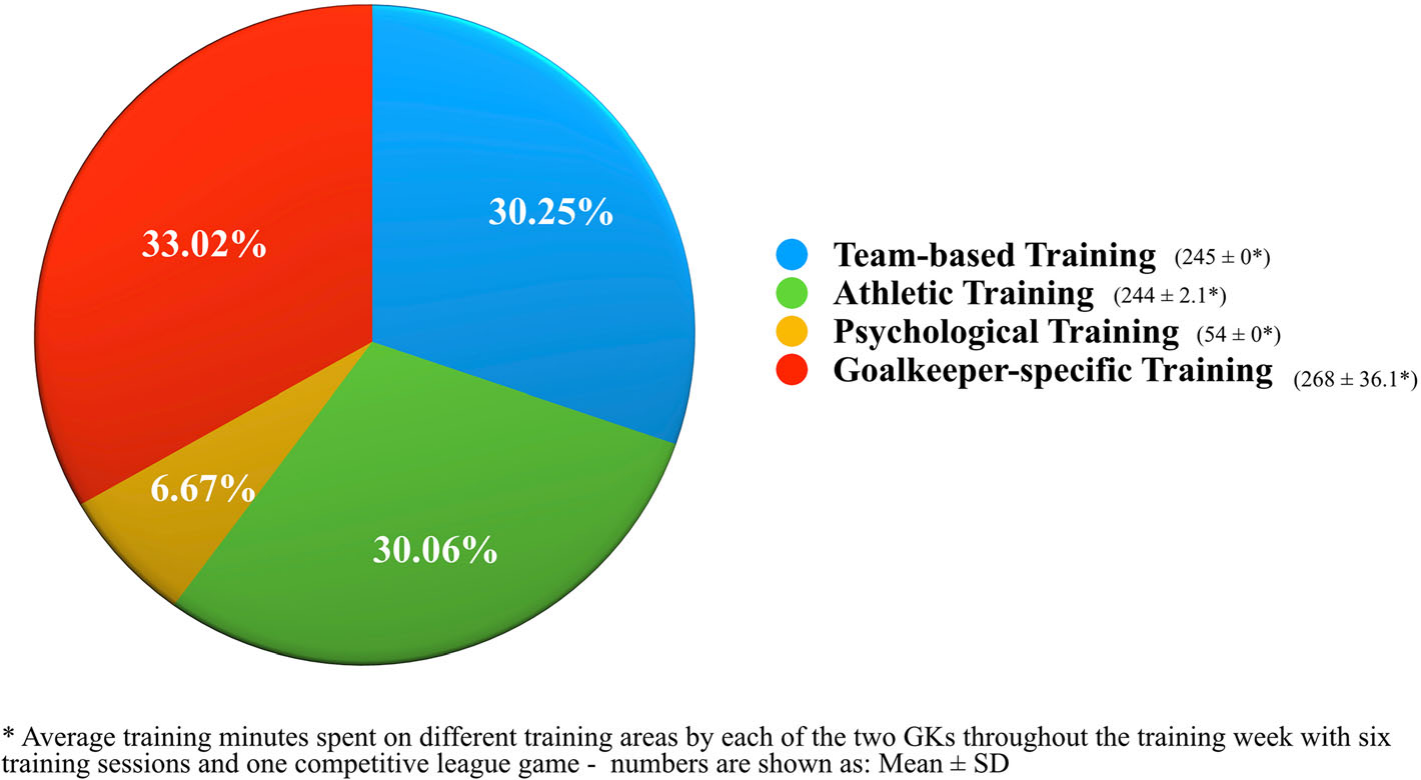
Example of GK training documentation including the average distribution of training time spent on various areas (i.e., team-based training, athletic training, psychological training and goalkeeper-specific training) for two GKs throughout the training week. Notably, the goalkeeper-specific training parts were co-designed with further members of the coaching staff (i.e., head coach, assistant coaches and performance analysts), partly incorporated outfield players and were tailored towards the team-based training parts

The case study of football goalkeeper (GK) training is drawn from a professional club in Europe during the 2020 season and driven by previous research on skill acquisition in goalkeeping (see [22, 23]). To further highlight benefits of applying a DoM structure to intra-organisational player development processes, the case study outlines a week-long training and game programme for two professional U23s age group GKs (see [1] for a related GK case study and an elaboration on the U23s context). In detail, Fig. 2 displays each GK’s average training time distribution throughout the week (i.e., six training sessions leading up to professional competition). All training was planned in collaboration between professional coaches and staff and was based on a unified methodical rationale and overarching (skill) training periodisation principles, including an athlete-environment-centred coaching focus and the notion of co-designing non-linear and representative learning environments [1, 2].

**Fig. 3:**
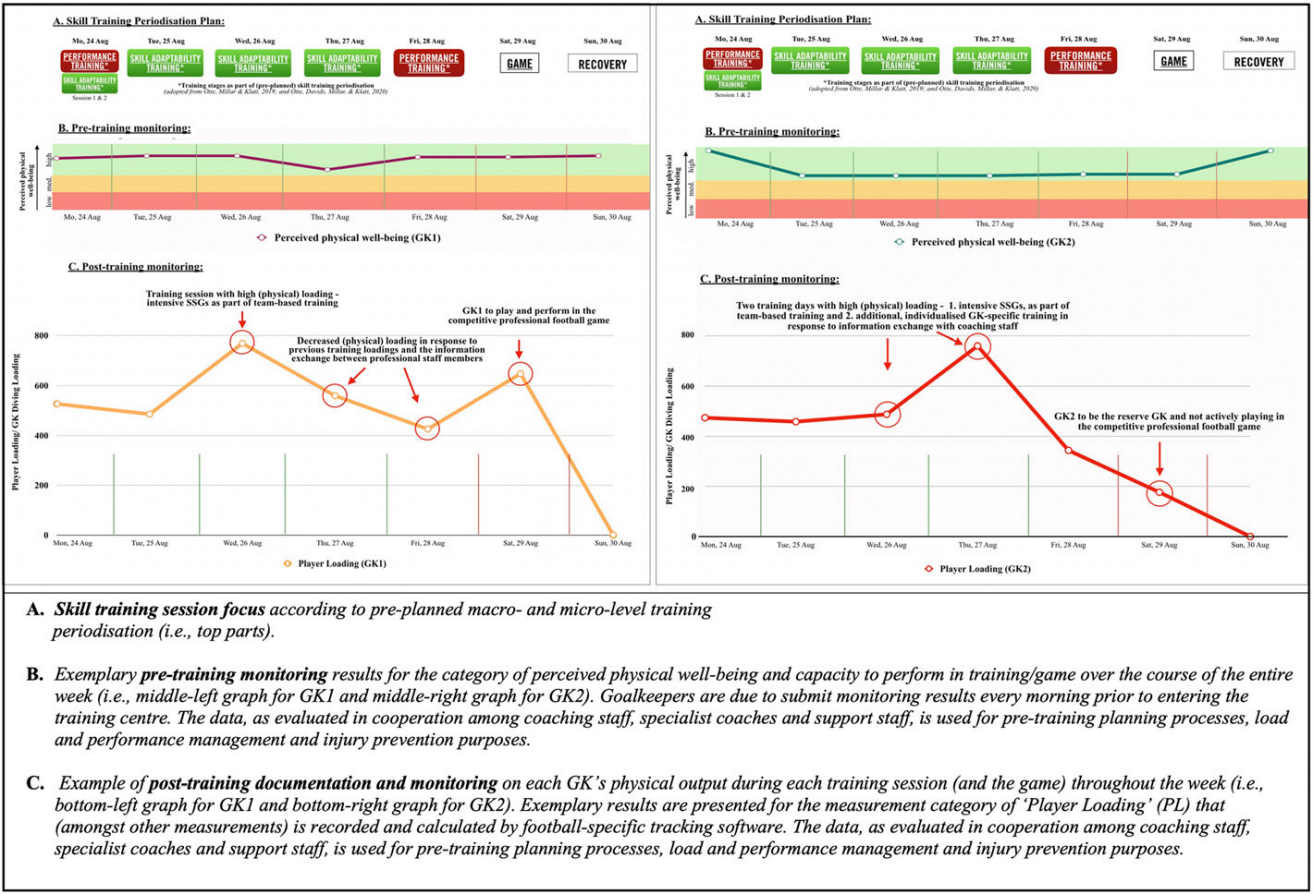
Annotated examples of pre- and post-training documentation including (**a**) the skill training focus according to a skill training periodisation plan (i.e., top part; see [22]); (**b**) the measurement of perceived physical well-being (i.e., the middle-left graph for goalkeeper 1 and middle-right graph for goalkeeper 2) and (**c**) the exemplary physical output data on each goalkeeper’s training loading (i.e., the bottom-left graph for goalkeeper 1 and bottom-right graph for goalkeeper 2). Notably, the data shown in the graphs is only representative of that usually collected by sport scientists and coaches in high-performance training environments

How does the training time split affect the roles and responsibilities of specialist coaches? Particularly, the two areas of *GK-specific training* and *team-based training* (i.e., accounting for more than 63% of all training time) outline the invaluable need for collaboration between head coaches, support staff and specialist coaches. In detail, this coordination between coaches appears critical in order to ensure facilitation of effective training designs (i.e., pre-planned on the micro-level for the week and on a day-to-day basis for single sessions [1]). Whilst football-specific training and the co-design of training sessions (i.e., integrating viewpoints from both coaching staff and specialists) have previously been conceptualised (see [2]), the exchange between specialists and support staff members (see Fig. 1) may warrant further elaboration. To provide an example of how co-design between specialists and sport scientists could work, Fig. 3 presents exemplary pre- and post-training documentation of (A) a pre-planned skill training periodisation plan for the week (i.e., top parts of Fig. 3); (B) athletes’ daily perceived feelings of well-being (i.e., as collected prior to training by the sports science and medical department; see central graphs in Fig. 3); and (C) the documentation of exemplary physical loading for trainings and competition (i.e., as, for example, obtained by specialist GK coaches through use of performance tracking software; see the bottom graphs in Fig. 3). Notably, as detailed elaboration of here-presented physical data would exceed the scope of this paper, please see annotations and descriptions in Fig. 3.

In order for professional staff members to adequately monitor, periodise and plan (physical and psychological) athlete loading and successive training sessions, multi-directional information exchange and communication about compiled data across all staff members become paramount. Linking this idea back to an overarching DoM, this case study reiterates one critical notion: the imperative need for coaching staff, specialist coaches (e.g. GK coaches) and support staff (e.g. sport scientists and psychologists) to co-design training and constantly exchange information.

### Case Study 3: Advancing Upfield in Rugby League

In Rugby League football, ‘metres gained’ and ‘making quick ground’ with the ball are important predictors of successful match outcomes [24, 25]. Traditionally, the performance challenge of advancing into opposition territory would be the sole responsibility of the Head Coach (tactical and technical specialist) to improve the team’s collective penetrative performance assessed by key performance indicators (KPIs). Specialist coaches (e.g. kicking coach) and support staff would also work with the players to address factors that are deemed important to improving these KPIs (i.e., physiological and psychological factors, perceptual awareness and tactical knowledge). Typically, however, these interventions would be delivered independently in a non-integrated manner, which could result in dissonance between the task constraints experienced in practice and competition.

To improve a team’s ability to gain metres onfield in possession of the ball, players could develop the physical, perceptual and emotional skills to collectively perceive and use relevant environmental opportunities offered to advance the ball forward. Therefore, all players must be afforded representative opportunities to perceive shared information sources in practice, which are related to physical and task constraints on action in competition [26]. However, these information sources are often not accurately represented in practice, where learning activities, such as decomposed practice tasks (i.e., the removal of attackers or defenders during team play) or small-sided games, are implemented without the careful consideration of tactical, physiological or psychological demands of competition [27]. To address this challenge, a DoM facilitated by all performance staff and key players in a heterarchical fashion would integrate the efforts of sub-discipline specialists to co-design representative learning tasks that sample relevant informational constraints under match play conditions. This could be achieved by the integrated efforts of (1) sport scientists using GPS data to identify in possession movement and physiological match demands [28], (2) a performance analyst identifying constraints on ball reception (e.g. distance of the receiver from the opposition, area of the pitch where the ball is received, kick chase defenders positioning and speed of chase and defence to attack transition actions) immediately before the team start to attack, (3) a strength and conditioning coach advising how to replicate in possession movement and physiological match demands during practice, (4) a specialist kicking coach guiding the search of all players to areas of the field where space may become available to kick the ball and subsequently pressuring those areas when transitioning to defence, and (5) a skill acquisition specialist identifying individual, sub-group and team selection pressures to support the enrichment of shared affordances and to ensure the representativeness of practice tasks [29]. Such an approach can strengthen a team’s self-organisation tendencies to achieve shared goals, such as efficiently and effectively gaining metres, by collectively perceiving and responding to environmental resources that invite shared actions.

## Conclusions

In this opinion piece, we proposed how a DoM could functionally integrate the work of specialist (role) coaches in coordinated, collective organisational collaborations, underpinned by the theoretical framework of ecological dynamics. This type of organisational design for support staff in high-performance sports organisations could empower specialist coaches to contribute meaningfully to individual athlete development and preparation for performance, without feeling marginalised. Indeed, further empirical work is needed to understand how such an integrated collective system of support staff can best enrich athlete development and engagement in the learning process across the course of their careers.

## Data Availability

Not applicable
